# Care Seeking Behaviour for Children with Suspected Pneumonia in Countries in Sub-Saharan Africa with High Pneumonia Mortality

**DOI:** 10.1371/journal.pone.0117919

**Published:** 2015-02-23

**Authors:** Aaltje Camielle Noordam, Liliana Carvajal-Velez, Alyssa B. Sharkey, Mark Young, Jochen W. L. Cals

**Affiliations:** 1 Maastricht University, School for Public Health and Primary Care (CAPHRI), Maastricht, the Netherlands; 2 Division of Policy and Strategy, Data and Analytics Section, United Nations Children Fund (UNICEF), New York, New York, United States of America; 3 Health Section, United Nations Children Fund (UNICEF), New York, New York, United States of America; St. Michael's Hospital, CANADA

## Abstract

Pneumonia is the leading cause of childhood mortality in sub-Saharan Africa (SSA). Because effective antibiotic treatment exists, timely recognition of pneumonia and subsequent care seeking for treatment can prevent deaths. For six high pneumonia mortality countries in SSA we examined if children with suspected pneumonia were taken for care, and if so, from which type of care providers, using national survey data of 76530 children. We also assessed factors independently associated with care seeking from health providers, also known as ‘appropriate’ providers. We report important differences in care seeking patterns across these countries. In Tanzania 85% of children with suspected pneumonia were taken for care, whereas this was only 30% in Ethiopia. Most of the children living in these six countries were taken to a primary health care facility; 86, 68 and 59% in Ethiopia, Tanzania and Burkina Faso respectively. In Uganda, hospital care was sought for 60% of children. 16–18% of children were taken to a private pharmacy in Democratic Republic of Congo (DRC), Tanzania and Nigeria. In Tanzania, children from the richest households were 9.5 times (CI 2.3–39.3) more likely to be brought for care than children from the poorest households, after controlling for the child’s age, sex, caregiver’s education and urban-rural residence. The influence of the age of a child, when controlling for sex, urban-rural residence, education and wealth, shows that the youngest children (<2 years) were more likely to be brought to a care provider in Nigeria, Ethiopia and DRC. Urban-rural residence was not significantly associated with care seeking, after controlling for the age and sex of the child, caregivers education and wealth. The study suggests that it is crucial to understand country-specific care seeking patterns for children with suspected pneumonia and related determinants using available data prior to planning programmatic responses.

## Introduction

Acute respiratory infections (ARIs) are the most common illnesses in childhood, of which lower respiratory tract infections (LRTIs) are the most severe in developing countries [1]. Pneumonia, a common and severe LRTI, was responsible for 15% of all deaths among children under-five in sub-Saharan Africa (SSA) in 2013 and most of these deaths were concentrated in a few countries [2, 3, 4]. Cough and fast and/ or difficult breathing (i.e. tachypnea and/or dyspnea) due to a problem in the chest are clinically recognized as signs of childhood pneumonia [5].

Effective antibiotic treatment for pneumonia exists, and therefore timely recognition of these signs and symptoms by primary caregivers and subsequent care seeking for treatment from ‘appropriate’ providers can prevent many of these deaths [6]. Nevertheless, only 50% of children in SSA with suspected pneumonia were taken for care in 2010 [7]. Caregivers may not seek care for myriad reasons: both financial (e.g., the cost of services or treatment, transportation costs, loss of wages) and non-financial (e.g., gender and social norms, insufficient knowledge of danger signs and illness severity, and previous experiences with health services) [8, 9, 10, 11]. Further analysis of care seeking behaviours by primary caregivers, and on child, caregiver and household characteristics associated with care seeking is needed to further optimise future strategies within integrated approaches to prevent and treat childhood pneumonia [12].

We examined care seeking behaviour by caregivers of children under-five years of age with suspected pneumonia in sub-Saharan countries with high rates of childhood pneumonia mortality, and examined to what extend caregivers and household characteristics influenced care seeking.

## Methodology

### Data sources

We analysed data from the Demographic and Health Surveys (DHS) or Multiple Indicator Cluster Surveys (MICS) of countries in sub-Saharan Africa identified by the Global Action Plan for Pneumonia and Diarrhoea (GAPPD) as being among those with the highest burden for pneumonia [12]. DHS and MICS surveys are typically conducted by government statistics agencies every 3 to 5 years, with the support and technical assistance of the United States Agency for International Development (USAID) and the United Nations Children's Fund (UNICEF) respectively. Both surveys are relatively similar in content and scope and have comparable results. DHS and MICS survey programs enable low-and middle-income countries (LMICs) to produce estimates of a range of indicators in the areas of health, education, child protection and HIV & AIDS. Leading agencies designing and implementing these surveys (USAID and UNICEF) work together to harmonize tools and methods to enable comparisons of key indicators across countries and over time. Both surveys adhere to the fundamentals of scientific sampling, including complete coverage, suitable sample sizes, pre-selection of sample households, and sample documentation. However, limitations due to cost or other practical considerations, such as security, might result in some inconsistencies [13]. These survey data are available publically at http://www.dhsprogram.com and http://www.data.unicef.org respectively.

Countries were considered eligible for inclusion in this analysis if: (1) a population-based survey was conducted during or after 2010, (2) the data was available at the start of our analysis (June 2013) and (3) the survey included the standard question on care seeking for children under-five with suspected pneumonia (cough and rapid or difficulty breathing due to a problem in the chest) and whether or not the caregiver sought care and from where they sought care during the past two weeks.

Questions relating to child’s health are included in the women’s questionnaire in DHS and in the questionnaire for under-fives in MICS. In DHS, only mothers were interviewed, while in MICS the under-five questionnaire is administered to either mothers or primary caregivers of children under-five.

The following questions (similar in DHS and MICS) were used for the analysis:

1. Has (NAME) had an illness with a cough at any time in the last 2 weeks? (Yes/ No/ Don’t know)
2. When (NAME) had an illness with a cough, did he/she breathe faster than usual with short, rapid breaths or have difficulty breathing? (Yes/ No/ Don’t know)
3. Was the fast or difficult breathing due to a problem in the chest or to a blocked or runny nose? (Problem in chest only/ Blocked or runny nose only/ Both/ Other (*specify*)/ Don’t know)
4. Did you seek advice or treatment for the illness from any source? (Yes/ No/ Don’t know)
5. A. Where did you seek advice or treatment? B. Anywhere else? (Various services which fall under the following categories: Public sector, Private sector, Other sources and Other (*specify*))

In order to be included in this analysis, the respondent had to answer “yes” to questions 1, 2 and 4, and ‘a problem in the chest’ to question 3. Multiple answers were possible for question 5 (sections A and B).

### Method of analysis

We used recode manuals and guides from both DHS and MICS prior to analysis and recoding, which was conducted using STATA 12.1. We weighted the data according to sample size by using the samples weight variables (V005 in DHS and chweight in MICS) provided in the dataset and explained in dataset guides. In case of missing data, the cases were excluded from the analysis. Data analysis was cross-checked by an independent researcher.

To analyse the rate of care seeking for pneumonia, we first calculated how many children under-five had suspected pneumonia (cough and rapid or difficulty breathing due to a problem in the chest), and then the percentage of those taken for care. For those taken for care, we then categorised them into health providers, also known as ‘appropriate’ providers (i.e., accredited by that country’s government authorities) or other provider also known as ‘non-appropriate’ providers (i.e., those not accredited to provide antibiotics). These other providers, for this analysis, include private pharmacies, shops, and traditional healers amongst others, for a more detail see the subsection below ‘categorisation of health care providers and facilities’. In both surveys caregivers can report more than one source of care to which they brought their child with suspected pneumonia during the past two weeks.

We calculated frequencies and cross tabulations and performed chi-square (χ2) tests to identify variables associated with the dependent variable ‘care seeking behaviour from an ‘appropriate’ provider’. To determine the adjusted associations between child and caregiver characteristics and care seeking behaviour, we predefined the following groupings of independent variables: child’s age (grouped as < 2 years and 2–5 years), child’s sex (male, female), residential setting (urban, rural), primary caregiver’s or mother’s education (none, primary, secondary and higher) and household wealth quintile (poorest, poorer, middle, richer and richest), which is a composite measure of a household’s cumulative living standard used by DHS and other surveys in countries that lack reliable data on income and expenditures [14]. We selected ‘appropriate’ providers as the dependent variable as they have undertaken formal training and are accredited to provide antibiotics and for that reason, we wanted to assess which factors are associated with seeking care from these providers. We performed a multivariate logistic regression model for the dependent variable in order to examine independence of associations (p ≤ 0.05), with all predefined variables in the model. In addition, we calculated odds ratios (ORs) with corresponding 95% confidence intervals (CIs).

### Categorisation of health care providers and facilities

When we refer to ‘any provider’, this includes all possible care providers (i.e. both ‘appropriate’ and ‘non-appropriate’ providers).

The definition of health providers, also known as ‘appropriate’ providers (and therefore referred to as such throughout this paper), varies among countries. However, this category includes both private and public providers who have undergone formal training and facilities that have received accreditation and are therefore authorized to treat children with signs of ARI, for example:

- Hospitals: For all countries this includes private and government hospitals.
- Primary health care (PHC) facilities: This category includes both private and public health centres, clinics, dispensaries and posts and in some cases a private doctor or other medical personal, village/ community health workers, mobile (outreach) services as well as health facilities supported by non-governmental organizations (NGOs).
- Unidentified other services: These are other government led facilities; however, they are not specified in the country specific databases.

Other providers, also known as ‘non-appropriate’ providers (and therefore referred to as such throughout this paper): include providers that are neither accredited nor authorized to prescribe antibiotics for children with signs of ARI. This varies by country, for this analysis, it includes private pharmacies, shops, and markets as well as relatives and those practicing traditional medicine or faith healing. It also includes private services which are unidentified in the country specific databases.

## Results

Of the ten highest burden countries initially considered for the analysis; six met the predefined inclusion criteria of having a DHS or MICS survey in 2010 or later (with data publically available for analysis) and including the standard indicator on suspected pneumonia and care seeking: Burkina Faso, Democratic Republic of Congo (DRC), Ethiopia, Nigeria, Tanzania and Uganda, see Table 1. Together, these six countries account for 297,000 deaths or 53% of childhood pneumonia mortality among children under-five in sub-Saharan Africa, and 26% of global childhood pneumonia mortality [15]. The remaining four countries were excluded for the following reasons: Angola did not have a survey assessing the indicators of interest available, Kenya did not have a survey in 2010 or later and relevant survey data for Niger and Mali were not available at the time we started our analysis. Table 1 shows the under-five mortality per 1,000 live births based on the latest estimates developed by the UN Inter-agency Group for Child Mortality Estimates [4]. The table also presents characteristics of the surveys in the included countries. Data of 76530 children were available for analysis. Sample sizes per country ranged from 7535 (Uganda) to 25192 (Nigeria).

**Table 1:** Countries included in the analysis, data source and year, and sample size of respondents and children with suspected pneumonia.

Country	Data source	Survey Year	Under-five mortality per 1000 live births	Children included in the survey	Children included in survey with suspected pneumonia		Children with suspected pneumonia from whom care was sought from an ‘appropriate’ provider	Children with suspected pneumonia from whom no care was sought
					Number [missing]	Percentage		
Burkina Faso	DHS	2010	98	14001	261 + [6]	1.9%	57.1%	29.9%
DRC	MICS	2010	119	11093	700 + [0]	6.3%	40.3%	34.7%
Ethiopia	DHS	2011	64	11042	767 + [6]	7.0%	27.2%	70.5%
Nigeria	MICS	2011	117	25192	890 + [0]	3.5%	39.7%	37.7%
Tanzania	DHS	2010	52	7667	326 + [6]	4.3%	72.0%	15.0%
Uganda	DHS	2011	66	7535	1115 + [3]	14.8%	78.9%	16.4%

Data on the under-five mortality rate are deaths per 1000 live births, and based on the 2013 estimates developed by the UN Inter-agency Group for Child Mortality Estimation; these modelled estimates rely on the quality of the underlying data [4]. For the numbers from the surveys, all numbers are rounded, adjusted for sample size (weighted) and missing data. For care seeking, all children are included who had suspected pneumonia and who sought care at least once.

### Care seeking for suspected *pneumonia*

The number of children under-five included in the survey with suspected pneumonia, as reported by caregivers in the previous 2 weeks preceding the surveys, ranged from 267 in Burkina Faso to 1118 in Uganda in the adjusted country samples. In all countries, except Ethiopia, care was sought for the majority of children with suspected pneumonia.

**Fig 1:**
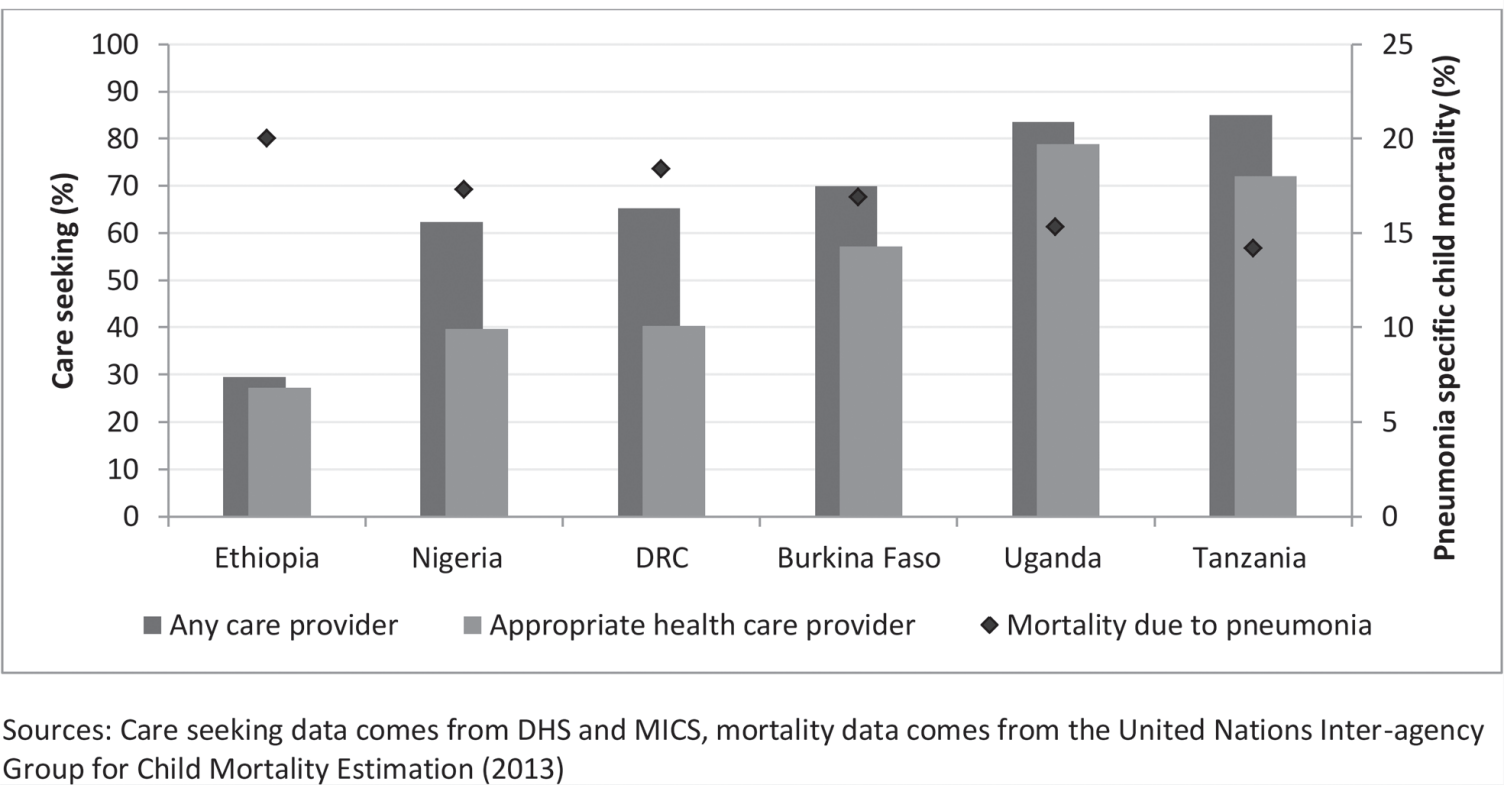
Care seeking for signs of childhood ARI by provider type, and overall level of pneumonia specific child mortality by country.

Fig. 1 shows the large variation in care seeking patterns for suspected pneumonia found across the six countries, juxtaposed with overall levels of pneumonia mortality. In Tanzania 85% of children with suspected pneumonia were taken to a provider by their caregiver, as opposed to only 30% in Ethiopia.

Differences are also seen across the countries in overall levels of care seeking from ‘appropriate’ providers (i.e. health care providers), ranging from 27% in Ethiopia to 79% in Uganda. In both these countries, when people do seek care they most often use ‘appropriate’ providers. Uganda and Tanzania were the only two countries where more than 70% of children were brought to an ‘appropriate’ provider. In Nigeria and DRC, overall care seeking is just over 60%, but care seeking from ‘appropriate’ providers stands at or just below 40%. All six countries present relatively high levels of pneumonia specific mortality.

Among only those children for whom care was sought, Table 2 shows to which specific type of health care facilities and providers these children were brought. In Ethiopia, Tanzania and Burkina Faso children were most often taken to a primary health care (PHC) facility, which includes health centres, clinics and posts as well as community based and outreach services. Hospitals were also frequently accessed for care in Nigeria and Uganda; in fact, in Uganda children were more likely to be taken to a hospital than a PHC facility. With respect to ‘non-appropriate’ or other providers, private pharmacies are frequently accessed in DRC, Tanzania and Nigeria (18, 18 and 16% respectively). Traditional practitioners and other providers (e.g. vendors, shops, churches, relatives or friends) are also often consulted in DRC and Nigeria in particular.

**Table 2:** Total number of visits to each facilities and/or providers within the six countries (%).

		**Burkina Faso**		**DRC**		**Ethiopia**		**Nigeria**		**Tanzania**		**Uganda**	
		**n**	**%**	**n**	**%**	**n**	**%**	**n**	**%**	**n**	**%**	**n**	**%**
**Total % of children brought for care**		**183**	**70.1**	**457**	**65.3**	**226**	**29.5**	**555**	**62.3**	**277**	**85.0**	**932**	**83.6**
**Total visits to any provider**		**188**	**100**	**520**	**100**	**245**	**100**	**596**	**100**	**296**	**100**	**999**	**100**
**Total visits ‘appropriate’ care (i.e. health) provider**		**149**	**79**	**294**	**57**	**225**	**92**	**369**	**62**	**244**	**82**	**938**	**94**
	Hospital	38	20	62	12	14	6	174	29	43	15	596	60
	PHC facility	111	59	175	34	211	86	152	26	201	68	339	34
	- Health center	110	59	103	20	113	46	66	11	59	20	-	-
- Clinic	-	-	-	-	71	29	-	-	-	-	301	30
- Post	-	-	52	10	20	8	23	4	-	-	-	-
- Dispensary	-	-	-	-	-	-	-	-	142	48	-	-
	- Private medic	-	-	4	1	-	-	11	2	-	-	12	1
- Community health worker	1	1	15	3	0	0	33	6	-	-	18	2
- Mobile (outreach) clinic	-	-	1	0	-	-	19	3	-	-	0	0
	- NGO services	-	-	-	-	7	3	-	-	-	-	-	-
Unidentified other	-	-	57	11	0	0	43	7	-	-	11	1
**Total visits ‘non-appropriate’ (i.e. other) providers**		**39**	**21**	**226**	**43**	**20**	**8**	**227**	**38**	**52**	**18**	**61**	**6**
	Private Pharmacy	13	7	93	18	14	6	94	16	52	18	19	2
	Traditional Practitioner	15	8	31	6	2	1	40	7	-	-	7	1
	Any other^a^	11	6	102	20	4	2	93	16	-	-	35	4

As some of the children were taken to more than one care provider, each visit is included separately (e.g. if a child was taken to a private *and* government hospital, both visits are included under hospital), the numbers are therefore higher than as the number of children brought for care. Numbers are rounded. Data is adjusted for sample size.

^a^ Includes all visits to any other ‘non-appropriate’ provider such as a vendor, shop, church, relative or friend and any other un-identified ‘non-appropriate’ provider.

Table 3 shows the results of the multivariate logistic regression to examine potential associations between child or caregiver characteristics and care seeking behaviour from ‘appropriate’ providers. In Nigeria, the influence of the age of a child, when controlling for the other variables (i.e. sex, urban-rural residence, education and wealth) shows that younger children (<2 years) were 1.7 times more likely to be brought to a care provider than children between 2 and 5 years of age (95% CI = 1.1–2.5, p<0.01). This significant association between younger age of the child and care seeking from ‘appropriate’ providers was also found for Ethiopia and DRC. In general, the influence of a caregiver’s education, when controlling for other variables shown in Table 3 shows that higher educated caregivers and higher wealth quintiles were also associated with higher levels of care seeking from ‘appropriate’ providers in this group of countries. In Uganda having at least a primary education, when controlling for age and sex of the child, urban-rural residence and wealth, was associated with higher likelihood to seek care. In Tanzania, Ethiopia, Nigeria and Burkina Faso, caregivers from the richest quintile were much more likely as those from the poorest quintile to seek care for their child with suspected pneumonia, after controlling for age and sex of the child, urban-rural residence and education, with odds ratios ranging from 4.7 (95% CI 1.5–15.1) to 9.4 (95% CI2.3–39.3). Similar patterns did not emerge in the DRC and Uganda. With regard to the sex of a child, controlling for the child’s age, urban-rural residence, education and wealth, girls were 1.7 times more likely to be brought to seek care in Uganda than boys (95% CI = 1.2–2.4, p<0.05). We found no statistically significant differences in care seeking patterns between rural and urban settings after controlling for the age and sex of a child, education and wealth.

**Table 3:** Associations among key child and caregiver characteristics and care seeking from any ‘appropriate’ provider.

Country	Burkina Faso		DRC		Ethiopia		Nigeria		Tanzania		Uganda	
Variable	N (%)	OR + (95% CI)	N %	OR + (95% CI)	N %	OR + (95% CI)	N %	OR + (95% CI)	N %	OR + (95% CI)	N %	OR + (95% CI)
**Age:**												
< 2 years	83/135 (61.2)	1.4 (0.8–2.4)	172/379 (45.3)	1.5 (1.0–2.3)*	117/361 (32.5)	1.8 (1.1–2.8)*	186/418 (44.6)	1.7 (1.1–2.5)**	118/153 (77.1)	1.5 (0.8–3.0)	435/553 (78.7)	1.0 (0.7–1.4)
2–5 years	66/126 (52.7)	ref	111/321 (34.5)	ref	91/406 (22.5)	ref	167/473 (35.4)	ref	117/172 (67.6)	ref	445/562 (79.1)	ref
**Sex of child:**												
Male	78/146 (53.3)	0.8 (0.5–1.4)	143/386 (36.9)	0.8 (0.5–1.2)	100/393 (25.4)	0.8 (0.5–1.2)	184/474 (38.8)	0.9 (0.6–1.4)	128/170 (74.9)	1.4 (0.7–2.6)	433/578 (74.9)	0.6 (0.4–0.8) **
Female	71/114 (62.0)	ref	140/314 (44.6)	ref	109/375 (29.1)	ref	170/416 (40.8)	ref	107/155 (69.0)	ref	447/537 (83.3)	ref
**Residence:**												
Urban	46/71 (65.5)	0.8 (0.3–1.8)	66/160 (41.4)	0.8 (0.4–1.6)	32/69 (46.9)	0.8 (0.3–2.0)	101/191 (53.0)	1.1 (0.6–2.0)	74/85 (86.1)	1.2 (0.4–3.2)	114/141 (80.8)	0.9 (0.4–1.9)
Rural	103/190 (54.0)	ref	216/540 (40.0)	ref	176/698 (25.2)	ref	252/699 (36.1)	ref	161/240 (67.1)	ref	766/974 (78.7)	ref
**Education:**												
Secondary +	*15/22 (—)*		87/206 (42.1)	1.2 (0.6–2.2)	*18/22 (—)*		124/214 (57.9)	1.6 (0.9–2.7)	22/26 (83.4)	1.0 (0.2–6.0)	164/201 (81.6)	1.9 (1.0–3.7)*
Primary	*25/37. (67*.*0)*	1.3 (0.5–3.3)	131/320 (40.8)	1.1 (0.7–1.9)	55/198 (27.9)	0.9 (0.5–1.6)	57/140 (40.8)	1.2 (0.8–2.0)	173/ 238 (72.8)	1.2 (0.6–2.7)	603/752 (80.2)	1.8 (1.1–2.8)*
No education	109/202 (54.2)	ref	65/174 (37.4)	ref	136/547 (24.8)	ref	173/535 (32.3)	ref	40/62 (64.7)	ref	113/162 (69.7)	ref
**Wealth index quintile:**												
Richest	46/66 (70.3)	4.7 (1.5–15.1)**	47/105 (44.7)	1.8 (0.7–4.5)	42/67 (61.8)	9.2 (3.1–27.0)**	67/90 (74.5)	6.0 (2.3–15.7)**	54/58 (93.4)	9.5 (2.3–39.3)**	140/170 (82.3)	1.2 (0.6–2.8)
Richer	37/65 (56.7)	2.4 (0.9–5.9)	48/115 (40.0)	1.3 (0.6–2.8)	57/173 (33.2)	2.7 (1.3–5.6)**	58/129 (45.0)	1.7 (0.8–3.5)	59/76 (77.6)	2.3 (0.8–7.0)	124/161 (77.2)	0.8 (0.5–1.5)
Middle	30/46 (64.7)	3.0 (1.2–7.9)*	63/164 (38.5)	1.3 (0.7–2.4)	43/191 (22.7)	1.7 (0.8–3.3)	68/144 (47.2)	2.2 (1.2–3.8)**	48/83 (57.8)	1.0 (0.4–2.5)	148/190 (78.1)	0.9 (0.5–1.5)
Poorer	*23/48 (47*.*2)*	1.5 (0.6–4.0)	74/153 (48.1)	1.8 (1.0–3.3)	37/148 (25.2)	1.9 (0.9–3.9)	79/233 (33.9)	1.4 (0.8–2.3)	40/48 (81.6)	3.2 (1.2–8.5)*	207/261 (79.2)	1.0 (0.6–1.6)
Poorest	*13/35. (36*.*8)*	ref	53/164 (32.4)	ref	29/188 (15.5)	ref	82/294 (27.8)	ref	35/61 (57.0)	ref	261/334 (78.3)	ref
**Total < 5 years**	**149/261 (57.1)**		**282/700 (40.3)**		**209/767 (27.2)**		**354/ 890 (39.7)**		**235/326 (72.1)**		**880/1115 (79.0)**	

All calculations: numbers (n), percentages (%), odds ratio’s (OR) and 95% confidence interval (CI) are based on weighted averages which are also adjusted for missing data. Numbers presented in this table are rounded, we used STATA for these analysis. Manually re-calculation might, therefore show slight differences.

*N (—)* less than 25 un-weighted cases

*N (%)* based on 25–49 un-weighted cases

Statistical significance: *p<0.05, **p<0.01

## Discussion

We found considerable variation in care seeking behavior for suspected pneumonia across six high pneumonia mortality countries in sub-Saharan Africa. The three countries found to have the lowest levels of care seeking from ‘appropriate’ providers (i.e. health facility/ provider) were Ethiopia, Nigeria and DRC.

### Overall care seeking

Country variations in care seeking for childhood infections, including pneumonia, have been reported previously [16, 17]. We concur with the conclusions of the Hodgins study [16] that programs should be adjusted to country specific needs based on identified barriers. Our data clearly show that both care seeking from ‘appropriate’ providers and childhood pneumonia mortality is high in Uganda, suggesting that the quality of care available to these children may be sub-standard or that children present for care late, as has been reported in other studies [18, 19, 20].

In Ethiopia, where we found care seeking to be the lowest among the six countries, strategies that investigate what the key challenges are related to accessing care need to be prioritized. Previous studies conducted in Ethiopia indicate that lack of knowledge and delay in recognition of the severity of an illness are important factors that predict care seeking [21, 22], as are religion [23] and household wealth [24]. Further, vast distances to facilities within the country were the impetus for the health extension worker program, which has made important strides in increasing coverage of treatment for childhood illnesses, although some challenges remain [25]. Our analysis confirms that there is a strong association between wealth and care seeking in Ethiopia, which we also found in Tanzania, Nigeria and Burkina Faso.

Other studies report that the associations between wealth and care seeking are not only linked to whether care is sought or not, but also from which facility. Studies from Nigeria and Tanzania reported that poorer women are more likely to utilize facilities which provide poor quality services [26, 27]. In Tanzania, women living in rural areas tend to visit primary health care (PHC) facilities more often, whereas richer and higher educated women visit hospitals or better equipped health facilities. The main reasons for bypassing PHC facilities are related to the lack of diagnostic aids and drugs [28].

In each setting, once solutions to locally specific barriers in care seeking are identified, it will be critical to ensure that demand generation efforts are not jeopardized by sub-standard service and treatment availability [29, 30]. An earlier study that reported higher treatment rates in countries with well-established private sector services suggested that the appropriateness of treatments provided may be a challenge [17].

Our study also found high use of ‘non-appropriate’ providers (e.g. private pharmacies, traditional practitioners and other services, such as shops, churches, relatives, etc.) in both DRC and Nigeria, 43 and 38% of the total care seeking respectively (see Table 2). Another recent study from DRC indicates that while ‘formal care’ (in this paper referred to as health providers or ‘appropriate’ providers) is valued, the cost of services creates barriers and results in families either self-medicating or using traditional provider options [31]. Another study from DRC also concluded that costs were a barrier, but suggested that distrust of government health services is also a problem [32]. Similarly, studies of care seeking for childhood illnesses in Nigeria have reported high use of home care, drug vendors and private clinics due to financial constraints, wishing to try home management first, and poor recognition of the severity of the illness or waiting for the child to improve [33,34,35]. As Quinley & Govindasamy have reported, drug shops do often function as de facto clinics [36]. Strategies for improving the quality of care in these service delivery points may be an important program strategy to consider [16].

The only association between sex of the child and care seeking we found was in Uganda, where girls were actually more likely to be brought for care than boys. Earlier published findings from Asia indicate preferential care seeking for male children [37, 38], and previous African studies of the association between the sex of a child and care seeking in Tanzania, where no association was found [39, 40]. In addition, while studies have reported associations between better health outcomes and higher levels of caregiver education [34, 41], for associations between caregiver education and care seeking—hence increasing the likelihood of receiving correct treatment—we only observed an association in Uganda.

We found differences between care seeking in rural versus urban areas in Burkina Faso, Ethiopia, Nigeria and Tanzania, as has been reported in previous studies [21, 42]. For example, in Ethiopia the percentage seeking care from an ‘appropriate’ provider in rural areas is 25, in contrast to 47% in urban areas. However, when we controlled for wealth, education, sex and the age of a child, there was no independent association of urban-rural residence. This may be (partly) explained by lower education and wealth status of those living in rural areas. In relation to this, our analysis shows that lower wealth quintiles were associated with lower levels of care seeking from ‘appropriate’ providers in Burkina, Ethiopia, Tanzania and Nigeria, independent of the actual residency of caregivers. In other words, the poorest are the least likely to seek care, independent of where they live (i.e. in urban or rural settings). This implies that those living in rural areas are more disadvantaged due to poverty and access to health services. These associations were not found in DRC and Uganda.

Our study indicates that care seeking for children under the age of 2 with suspected pneumonia was more frequent than for children between 2–5 years of age, particularly in Nigeria, Ethiopia and DRC, this relationship was significant. Although similar findings were reported in previous studies from Nairobi [43] and Nigeria [44], studies from Uganda [19] and Ethiopia [24] reported no association between care seeking and age of the child. Seeking treatment for younger children with suspected pneumonia is especially critical to decrease childhood mortality due to pneumonia, not only because the incidence of pneumonia is highest amongst these children, but also because 81% of child deaths due to pneumonia occur within this age group [45]. Demand generation efforts should take this into account and should focus on preventive measures as well, e.g. increasing coverage of immunization, and improving breastfeeding practices and nutritional status [46, 47].

One of the strategies to improve the quality of care is through Integrated Management of Childhood Illnesses (IMCI)—a strategy designed to reduce child mortality and morbidity due to common illnesses. IMCI has been implemented in all 6 countries included in these analyses. The strategy aims to improve family and community health practices, case management of health staff and the overall health system [48], although the extent to which this is achieved in any particular setting will vary. The level of implementation, quality of training and supervision will have an impact on care seeking behavior and the quality of care [49, 50]. Moreover, the quality of care is not merely affected by the capacity of health workers, but also on the availability of essential resources, inkling appropriate medicines [49]. The IMCI protocol guides health workers to classify a child as having pneumonia when s/he presents with a cough and fast and/ or difficult breathing due to a problem in the chest. Despite the protocol, health workers are often challenged to classify and prescribe treatment for these suspected pneumonia cases [19, 51].

## Limitations

There are limitations related to these analyses. Pneumonia prevalence, which is collected in household surveys primarily for use as a denominator for indicators relating to pneumonia, should be interpreted with caution as it depends on caregivers’ perception of the signs and symptoms (which may or may not be accurate) and their capacity to recall the events (which may be prone to recall bias), leading to incorrect estimates [52]. Moreover, the prevalence of suspected pneumonia varies seasonally, which also influences care seeking (i.e., it may be more difficult to take a child for care during harvest and rainy seasons). In relation to this, caregivers may identify signs and symptoms such as cough and difficulty or rapid breathing due to a problem in the chest, while, clinically these may refer to another acute respiratory tract (ARI) infection, rather than to pneumonia specifically.

Secondly, survey data do not allow us to determine the specific pathways of care taken (i.e. which care provider—either ‘appropriate’ or ‘inappropriate’—was visited first), if the same provider was visited multiple times or if the same health worker assessed a sick child in a health center and again later in his or her capacity as a private pharmacist. Having this additional information would allow for more nuanced analyses of care seeking behaviors in these settings.

Finally, survey data also do not provide information on severity of illness, and it is therefore not possible to distinguish whether or not seeking care from PHC facility was appropriate, or if the child should have gone directly to hospital. In relation to this, it would be interesting to assess if a child received treatment, however, as Hazir et al (2013) concluded ‘…data in its current format from DHS/MICS surveys should not be used for the purpose of monitoring antibiotic treatment rates in children with pneumonia at the present time as their quality is jeopardized’ [52]. This is because the identified cases depend on the caregiver’s interpretation of signs and symptoms; the validity of receiving antibiotics is therefore dependent on the accuracy of this interpretation.

### Further research areas

In this analysis we included (when applicable) multiple providers/facilities per child, which could include both ‘appropriate’ and ‘inappropriate’ provider categories. Further research could assess the percentage of care sought from more than one type of provider and why. A better understanding of the complexity of care seeking and associated delays including the timing of care-seeking could reveal additional information about quality of care and user preferences. Further research should aim to understand the correlation between mortality and care seeking, and assess if there are differences in this association between any care seeking and care seeking from ‘appropriate’ providers. These analyses should focus not merely on pneumonia, but also on the other main causes of illnesses (e.g. diarrhea and fever) [53, 54]. Finally, we need to know why overall care seeking is unacceptably low in some countries, even for the youngest children (who benefitted from slightly higher levels of care seeking in this study but who are also the most likely to die from pneumonia). It is critical to identify strategies to improve the quality of services visited most often by caregivers, including the ‘informal’ sector, e.g. private pharmacies.

## Conclusion

In conclusion, this study illustrates that prior to planning strategies to decrease pneumonia mortality, it is crucial to understand care seeking patterns (and the related determinants) between countries and within countries using available data, such as national survey data. Further research is needed to better understand the reason behind these findings by conducting more systematic analyses at national and sub-national level, including the assessment of socioeconomic, knowledge and information, cultural and health system factors that influence care seeking. Locally specific research is also needed to understand why families choose the providers and facilities they choose, and then programmatic strategies should be developed that engage local community members to identify relevant, feasible and acceptable solutions.
