# Correction: Care Seeking Behaviour for Children with Suspected Pneumonia in Countries in Sub-Saharan Africa with High Pneumonia Mortality

**DOI:** 10.1371/journal.pone.0126997

**Published:** 2015-04-20

**Authors:** Aaltje Camielle Noordam, Liliana Carvajal-Velez, Alyssa B. Sharkey, Mark Young, Jochen W. L. Cals

The Data Availability statement for this paper is incorrect. The correct statement is: “DHS data can be obtained upon request at http://dhsprogram.com/data/new-user-registration.cfm. MICS data can be obtained upon request at http://mics.unicef.org/visitors/sign-up.”

In the Data sources subsection in the Methodology section, the fifth sentence is incorrect. Instead of reading, “Leading agencies designing and implementing these surveys (USAID and UNICEF) work together to harmonize tools and methods to enable comparisons of key indicators across countries and over time,” it should read: “DHS and MICS programs work closely together to harmonize tools and methods to enable comparisons of key indicators across countries and over time.”
